# Identification of immune-related features involved in Duchenne muscular dystrophy: A bidirectional transcriptome and proteome-driven analysis

**DOI:** 10.3389/fimmu.2022.1017423

**Published:** 2022-11-22

**Authors:** Xuan Wu, Nan Dong, Liqiang Yu, Meirong Liu, Jianhua Jiang, Tieyu Tang, Hongru Zhao, Qi Fang

**Affiliations:** ^1^ Department of Neurology, The First Affiliated Hospital of Soochow University, Suzhou, China; ^2^ Department of Neurology, Affiliated Hospital of Yangzhou University, Yangzhou, China

**Keywords:** Duchenne muscular dystrophy, transcriptome, proteome, immune, differentially expressed mRNAs, differentially expressed proteins

## Abstract

**Background:**

We aimed to investigate the biological mechanism and feature genes of Duchenne muscular dystrophy (DMD) by multi-omics and experimental verification strategy.

**Methods:**

We integrated the transcriptomic and proteomic methods to find the differentially expressed mRNAs (DEMs) and proteins (DEPs) between DMD and Control groups. Weighted gene co-expression network analysis (WGCNA) was then used to identify modules of highly correlated genes and hub genes. In the following steps, the immune and stromal cells infiltrations were accomplished by xCELL algorithm. Furthermore, TF and miRNA prediction were performed with Networkanalyst. ELISA, western blot and external datasets were performed to verify the key proteins/mRNAs in DMD patient and mouse. Finally, a nomogram model was established based on the potential biomarkers.

**Results:**

4515 DEMs and 56 DEPs were obtained from the transcriptomic and proteomic study respectively. 14 common genes were identified, which is enriched in muscle contraction and inflammation-related pathways. Meanwhile, we observed 33 significant differences in the infiltration of cells in DMD. Afterwards, a total of 22 miRNAs and 23 TF genes interacted with the common genes, including TFAP2C, MAX, MYC, NFKB1, RELA, hsa-miR-1255a, hsa-miR-130a, hsa-miR-130b, hsa-miR-152, and hsa-miR-17. In addition, three genes (ATP6AP2, CTSS, and VIM) showed excellent diagnostic performance on discriminating DMD in GSE1004, GSE3307, GSE6011 and GSE38417 datasets (all AUC > 0.8), which is validated in patients (10 DMD vs. 10 controls), DMD with exon 55 mutations, mdx mouse, and nomogram model.

**Conclusion:**

Taken together, ATP6AP2, CTSS, and VIM play important roles in the inflammatory response in DMD, which may serve as diagnostic biomarkers and therapeutic targets.

## Introduction

Duchenne muscular dystrophy (DMD) is a common clinical muscular dystrophy, which mostly occurs in males, accounting for about 1/3500 of live born boys (1). It is caused by a gene defect on the X chromosome, which encodes a mutated muscular dystrophy protein, a key component of the plasma membrane cytoskeleton (2). The disease usually characterizes by progressive weakness of skeletal muscle, atrophy and pseudohypertrophy of calf gastrocnemius muscle, and subsequently dies from respiratory insufficiency or cardiomyopathy (3). At present, there is no effective cure for DMD, and the commonly used treatment methods include cortisol hormone therapy (4). However, steroid therapy is accompanied by side effects such as obesity, dwarfism and osteoporosis, and cannot change the final outcome of the disease (5). Therefore, it is urgent to discover the new therapeutic targets and underlying mechanism, and identify biomarkers for prognostic evaluation.

Recently, increasing evidence has shown that transcriptomics and proteomics have been widely used to promote a better understanding of pathophysiological mechanisms and aid in diagnostic tool development in various disease (6–9). Some limited individual transcriptomic or proteomic studies have been performed to evaluate isolated histological, biochemical, and contractile changes in muscular dystrophy (10–12). Nevertheless, studies on integrating physiological measures with multiomic techniques are lacking. Furthermore, weighted gene coexpression network analysis (WGCNA) can describe the connections among various genes by constructing a co–expression network and identify phenotype-related modules, which is more effective to investigate the key pathways and genes in many human disorders (13, 14). In addition, most immune system components are demonstrated in the initiation and progression of DMD (15). Therefore, our goal was to perform a detailed analysis of molecular mechanisms, diagnostic and therapeutic targets underlying DMD with the transcriptome, proteome, and immune-related features.

**Figure 1:**
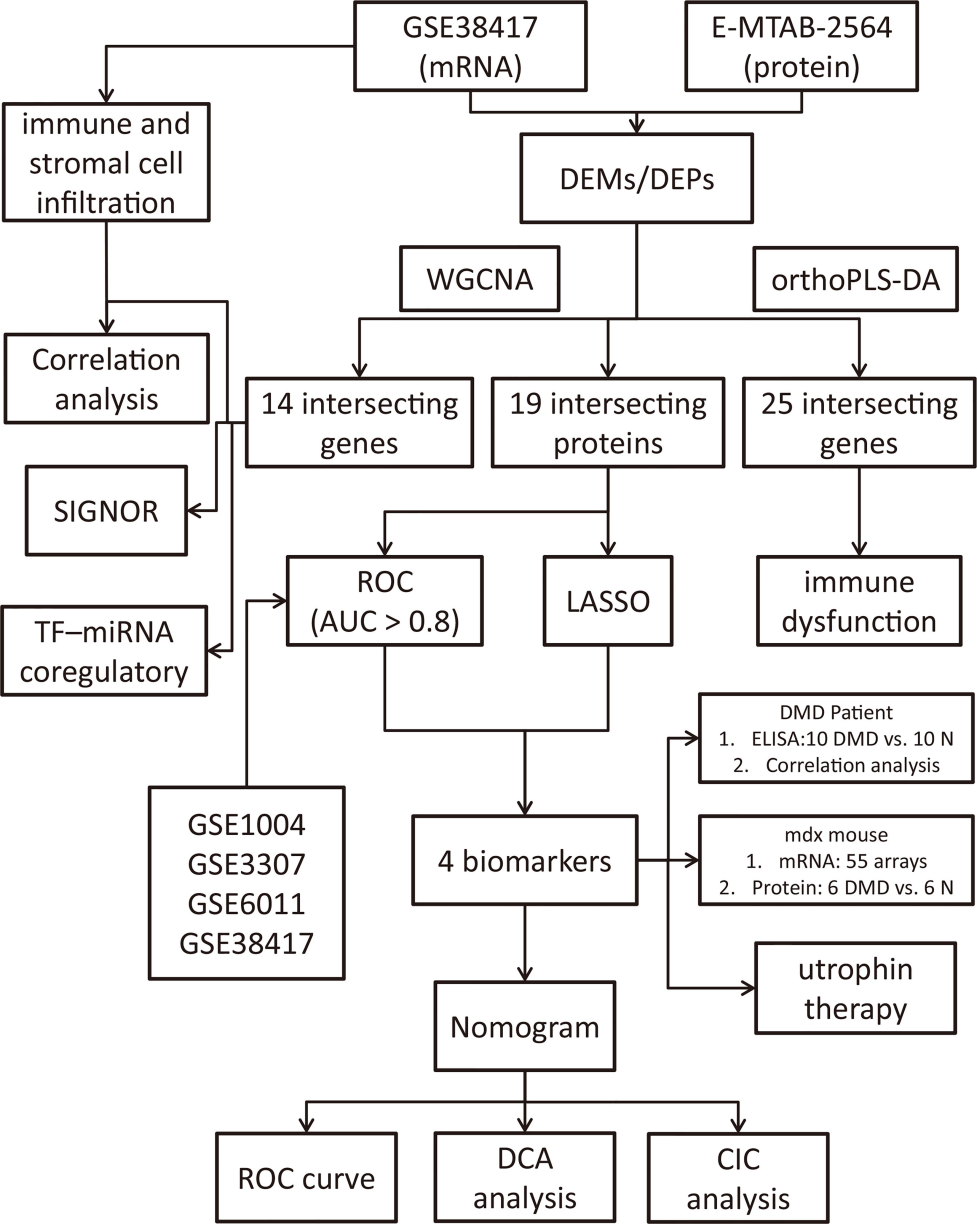
Flowchart of the study design.

In this study, we performed an integrated analysis to discovery functional genes concurrently involved in DMD across transcriptome and proteome and identified the shared signaling pathways of these intersecting genes. We found that multiple immune-related biological functions are dysregulated in both transcriptome and proteome, resulting in our subsequent exploration of immune-related intersecting genes, profiling of the immune cell proportion, and their shared relationships. Additionally, we explored the function and diagnostic value of the immune-related common genes and validate their potential clinical use as novel biomarkers and therapeutic implications in DMD patients and mdx mouse with machine learning and nomogram analysis. **Figure 1** showed the flowchart of the study design.

## Materials and methods

### Data acquisition from the GEO and ArrayExpress databases

DMD mRNA and protein expression data were obtained from Gene Expression Omnibus (GEO) (https://www.ncbi.nlm.nih.gov/gds) and ArrayExpress (https://www.ebi.ac.uk/arrayexpress/) databases. GSE38417 and E-MTAB-2564 were selected for discovery dataset. A total of 22 samples were collected from GSE38417 (mRNA), including 16 DMD and 6 normal skeletal muscle tissues. A total of 127 DMD and 58 normal plasma samples were collected from E-MTAB-2564 (protein). Then, we downloaded GSE1004 (12 DMD and 12 normal), GSE3307 (10 DMD and 18 normal) and GSE6011 (23 DMD and 14 normal) for validation dataset.

### Data preprocessing and differential expression analysis

Different intergroup comparisons were performed using the R software (v.3.6.0; https://www.r-project.org/). At first, the mRNA expression matrices of the GSE38417 and protein expression of E-MTAB-2564 datasets were normalized separately. Then, volcano plots and heatmaps of the differentially expressed mRNAs (DEMs) and proteins (DEGs) were plotted by https://www.bioinformatics.com.cn, a free online platform for data analysis and visualization. DEMs with |fold change| ≥ 1.5 and adjusted P-value <0.05 were considered to be statistically differentially expressed. DEPs with |fold change| ≥ 1.2 and P-value <0.05 were considered to be statistically differentially expressed. Moreover, orthogonal partial least squares - discriminant analysis (orthoPLS-DA) was performed to reduce the dimensionality and evaluate the independence of each group through MetaboAnalyst5.0 online tool (16).

### Weighted gene co-expression network analysis (WGCNA)

To identify modules of highly correlated genes and hub genes, we constructed scale-free co-expression networks for the GSE38417 and E-MTAB-2564 datasets separately through WGCNA (14). First of all, Pearson correlation-based hierarchical clustering was applied to cluster all the genes and samples. Then, soft threshold power value of network construction was selected to make the co-expression network in accordance with a scale-free co-expression network. Third, the adjacency matrices were transformed into a topological overlap matrix for gene modules recognition. In the following step, similar modules were clustered and merged. At last, the module-trait relationship diagrams were performed and the gene list of each module was acquired.

### Intersection of differential expression analysis and WGCNA

The top two modules, which are most closely related to DMD, were obtained through the module-trait relationship diagrams of the GSE38417 and E-MTAB-2564 datasets. Venn diagram analysis was further used to find overlapping genes between DEGs (DEMs and DEPs) and the above module genes, which were considered as candidate hub genes of DMD and applied for functional enrichment analysis through Metascape software (17).

### Profile of the immune and stromal cell infiltration

xCell (18) is a well-established method for calculating the immune and stromal cell composition of “bulk tissue” from microarray or RNA-seq data matrix, which includes 64 stromal and immune cell types. To obtain the differentially expressed cells in DMD, the cell enrich score of stromal and immune cells in the DMD samples were extracted and the difference was performed using Wilcoxon test. Ultimately, Pearson correlation test analysis was implemented to illustrate the relationship between hub genes and differential infiltrated immune and stromal cells, and |r| > 0.7 was set as a strong correlation threshold for further analysis.

### Signaling information network and TF-miRNA regulatory networks analysis of the hub genes

SIGNOR 2.0 (https://signor.uniroma2.it/) (19), a public repository that stores manually-annotated causal relationships between proteins and other biologically relevant entities, was performed to explore the signal transduction relationship of the former hub genes. Then, TF-miRNA coregulatory network analysis was implemented on the hub genes through curated regulatory interaction information collected from the RegNetwork repository (20) with Networkanalyst online tools (21).

### Screening process of the biomarkers for DMD

Intersecting proteins in DEPs and turquoise module from WGCNA were subjected to LASSO regression analyses in 127 DMD and 58 normal samples with glmnet package (22). The ten-fold cross-verification was employed to tune parameter selection. Lambda was used as the minimum partial likelihood deviance. Then, Receiver Operator Characteristic (ROC) curves were plotted to explore the sensitivity and specificity of the intersecting proteins for DMD diagnosis, which was validated in the other four validation datasets, including GSE1004, GSE3307, GSE6011 and GSE38417 datasets. AUC > 0.8 was set as an excellent diagnostic biomarkers threshold for further clinical and animal experiment validation.

### Validation of the biomarkers expressions in the DMD patients by ELISA

To further confirm the results of the proteomic analysis, four hub proteins were validated in 10 DMD patients and 10 healthy controls by ELISAs, including ATP6AP2 (Cat#F15798, YOYOBIO), CTSS (Cat#F08116, YOYOBIO), VIM (Cat#F11800, YOYOBIO), and TNFRSF1B (Cat#F08121, YOYOBIO). Since E-MTAB-2564 used plasma samples for screening biomarkers, we also collected plasma samples of all participants, as well as the characteristics of each patient (**Supplement Table 1**). Pearson’s correlation analysis was performed to analyze the correlation between the four proteins and the clinical characteristics, including age, white blood cell count (WBC), red blood cell (RBC), platelet count (PLT), C-reactive protein (CRP), alanine amiotransferase (ALT), aspartate aminotransferase (AST), hydroxybutyric dehydrogenase (HBD), creatine kinase (CK), creatine kinase-MB (CK-MB), lactate dehydrogenase (LDH), Motor Function Measure Scale (MFM) score and Mercuri score (23). This study was approved by the Medical Ethics Committee of the first affiliated hospital of Soochow University (No. 2020-145). Informed consent was obtained from all participants.

### Validation of the biomarkers expressions in mdx mouse and utrophin therapy groups

To further verify the above results, we examined the mRNA expression of the four hub genes of three hindlimb muscles (extensor digitorum longus, EDL; flexor digitorum brevis, FDB; soleus, SOL), comparing 2- and 5-month old WT and mdx mice to investigate degree and time-course of mdx pathology in GSE162455 dataset (55 samples). In addition, mdx mice on C57BL/10ScSnJNju background (mutation in exon 4 of the dystrophin) and wild type mice were bred and used (purchased from GemPharmatech Co. Ltd., n = 6/group). The protocols were approved by the Ethical committee from Soochow University. Experiments were conducted on males at 6–8 weeks of age. Dystrophin protein expression was quantified to evaluate the DMD modeling by immunofluorescence. Proteins were extracted from gastrocnemius muscle. Using SDS-PAGE, the proteins were separated and transferred to PVDF membranes. Incubation with the primary antibodies was carried out at 4°C overnight after blocking with 5% BSA. The secondary antibody was incubated for 60 min. The blots were visualized using the ECL Plus kit (NOBLEBIO, HP5002, Shanghai, China) and exposed to a ChemiDoc MP Imaging System (Bio-Rad, CA, United States). Protein density was calculated using Image Lab software. The variation of protein density was expressed as fold changes compared to the control in the blot. The antibodies used in this study were as follows: CTSS (Cat#A1874, 1:1000), VIM (Cat#A19607, 1:1000), ATP6AP2 (Cat#A6531, 1:1000), GAPDH (Cat#AF7021, 1:1000). Since utrophin is highly related to dystrophin and can substitute for dystrophin’s function (24), we explored the expression levels of these four genes in dystrophin-deficient mice with a transgene expressing high level of full length utrophin.

### Establishment and evaluation of a nomogram model

The “regplot” package was employed to build a nomogram model based on the three hub potential biomarkers. The calibration curve was further used to evaluate the predictive power of the model. Last but not least, decision curve analysis (DCA) and clinical impact curve (CIC) were employed to estimate the clinical value of the model.

## Results

### Identification of DEMs and DEGs

**Figure 2:**
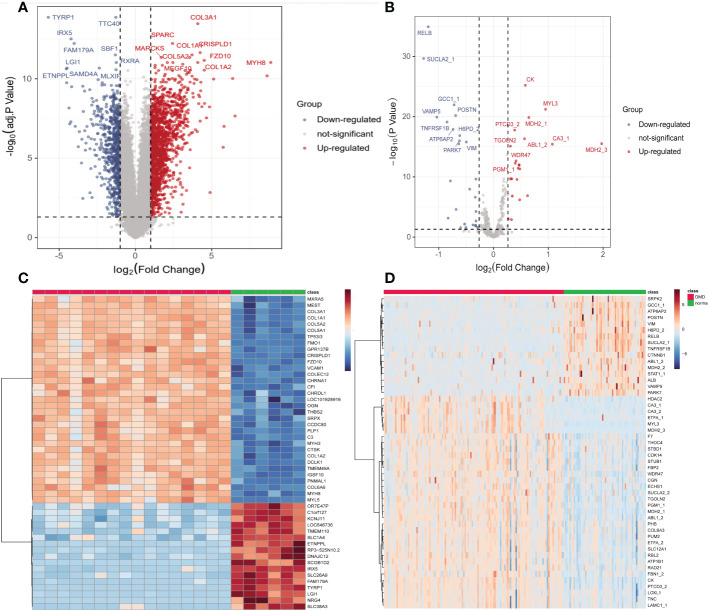
Identification of differentially expressed mRNAs (DEMs) and proteins (DEPs) in DMD. Volcano plots of DEMs in the GSE38417 **(A)** and DEPs in the E-MTAB-2564 **(B)** datasets. Heatmaps of the top 50 DEMs in the GSE38417 **(C)** and DEPs in the E-MTAB-2564 **(D)** datasets. Red, up-regulated DEMs/DEPs; blue, down-regulated DEMs/DEPs.

The GSE38417 dataset contained the gene expression matrix, including 16 DMD and 6 normal skeletal muscle tissues. The E-MTAB-2564 dataset contained the protein expression profiles from 127 DMD and 58 normal plasma samples. After data preprocessing and differential expression analysis, we identified 4515 DEMs from the GSE38417 dataset (|Fold change| ≥ 1.5 and adjust P value < 0.05); 2652 were up-regulated and 1863 were down-regulated, including MYH8, COL3A1, FZD10, TYRP1, IRX5 and FAM179A (**Figure 2A**). We also identified 56 DEPs from the E-MTAB-2564 dataset (|Fold change| ≥ 1.2 and P value < 0.05); 23 were up-regulated and 32 were down-regulated, including MDH2, MYL3, CA3, RELB, SUCLA2 and VAMP5 (**Figure 2B**). The top 50 DEMs(**Figure 2C**) and DEPs (**Figure 2D**) from each dataset were visualized in heatmaps.

### Orthogonal Partial Least Squares - Discriminant Analysis verifying independence of each group

**Figure 3:**
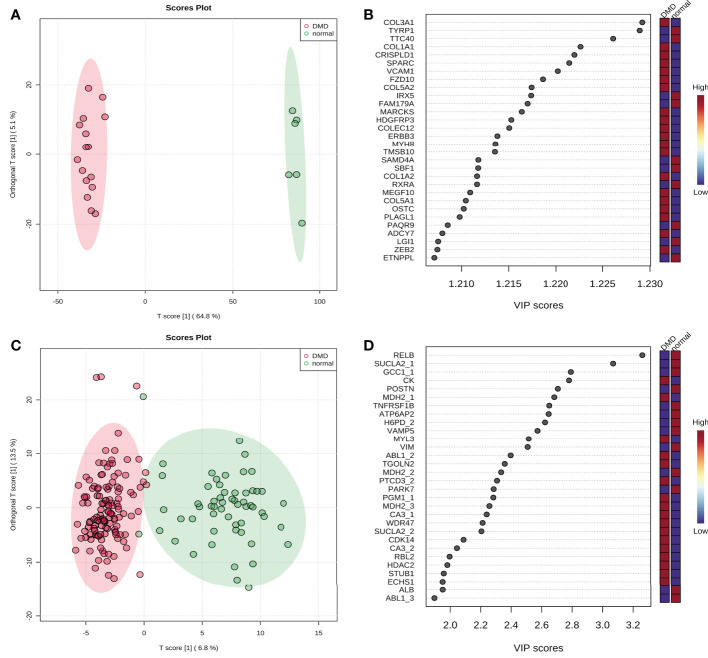
The score plot of Orthogonal Partial Least Squares - Discriminant Analysis. **(A)** GSE38417 dataset; **(B)** E-MTAB-2564 dataset; **(C)** the top 20 mRNAs according to VIP scores; **(D)** the top 20 proteins according to VIP scores.

**Figure 4:**
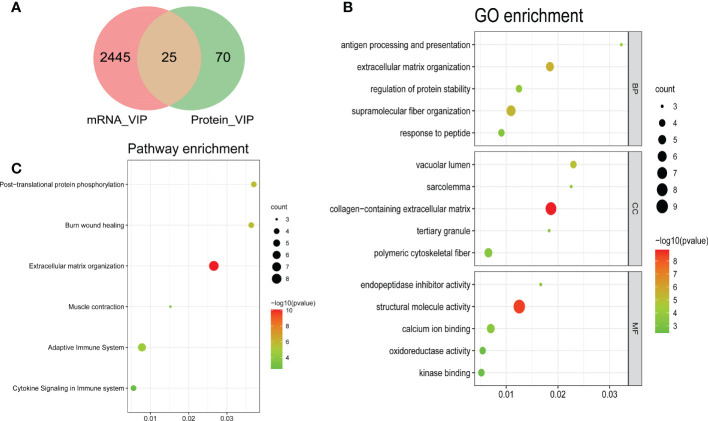
Bubble plots of the functional and pathway enrichment analyses of the overlapping genes. **(A)** A Venn plot showing the number of genes that have significant VIP scores in either mRNA level, protein level or both; Gene Ontology (GO) **(B)** and Kyoto Encyclopedia of Genes and Genomes (KEGG) pathway **(C)** enrichment analyses. The diameter of the circle indicates the number of genes, the color of the circle indicates the P-values, and GeneRatio represent the proportion of the total number of genes.

To distinguish the significant difference between normal and DMD samples, Orthogonal Partial Least Squares - Discriminant Analysis (orthoPLS-DA) was performed to reduce the dimensionality and evaluate the independence of each group. The results showed that normal samples vs. DMD samples in the two datasets displayed a significant difference (**Figures 3A, C**). In addition, VIP scores were used to distinguish the most important mRNAs and proteins between the groups. The top 10 important features of mRNA were COL3A1, TYRP1, TTC40, COL1A1, CRISPLD1, SPARC, VCAM1, FZD10, COL5A2 and IRX5. The top 10 important features of protein were RELB, SUCLA2, GCC1, CK, POSTN, MDH2, TNFRSF1B, ATP6AP2, H6PD and VAMP5 (**Figures 3B, D**). A total of 25 overlapping genes were detected between the DEMs and DEPs using VIP score ≥ 1(**Figure 4A**). GO enrichment analysis showed that these genes were mainly enriched in antigen processing and presentation, collagen−containing extracellular matrix, and endopeptidase inhibitor activity. Pathway enrichment analysis showed that these genes were mainly enriched in adaptive immune system, muscle contraction, cytokine signaling in immune system, and extracellular matrix organization, indicating immune dysfunction in DMD and were used for the subsequent analyses (**Figures 4B, C**).

### WGCNA of the whole transcriptome and proteome expression matrix

**Figure 5:**
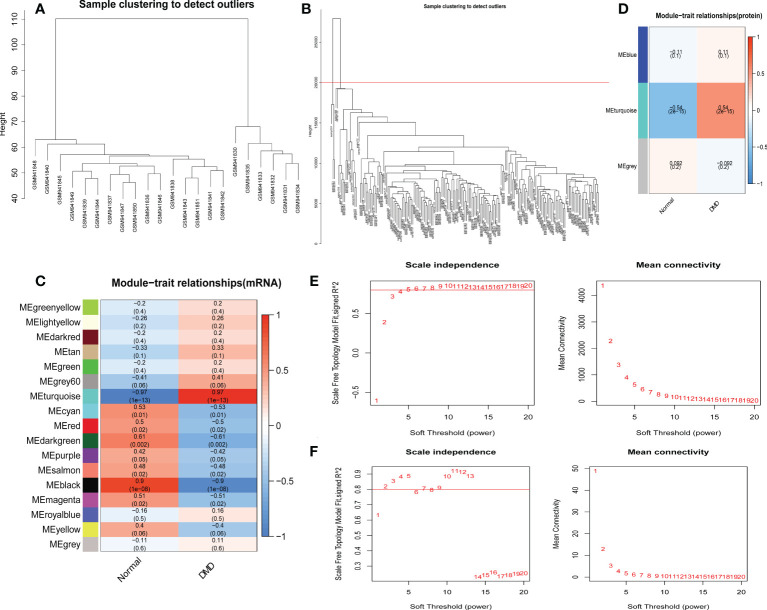
Identification of modules correlated with the clinical phenotype in the GSE38417 and E-MTAB-2564 datasets of DMD. Hierarchical clustering dendrograms of samples in the GSE38417 **(A)** and E-MTAB-2564 **(B)** datasets. Module-trait relationship diagrams in the GSE38417 **(C)** and E-MTAB-2564 **(D)** datasets. Each row corresponds to a color module and each column corresponds to a clinical trait (DMD or normal). Each cell contains the corresponding correlation and P-value. Analysis of the scale independence and mean connectivity for the optimal soft threshold powers in the GSE38417 **(E)** and E-MTAB-2564 **(F)** datasets.

Genes with similar expression patterns tended to exert similar biological functions. Therefore, we performed WGCNA according to the transcriptome and proteome expression matrix. The hierarchical clustering dendrograms of the samples are shown in **Figures 5A, B**. Then, we analyzed the soft threshold powers of the network topology and selected β = 5 and 3 as the optimal soft-thresholding parameters (**Figures 5E, F**). We identified 17 modules from the GSE38417 dataset (**Figure 5C**) and 3 modules from the E-MTAB-2564 dataset (**Figure 5D**), and plotted module-trait diagrams to explore the relationships between gene modules and DMD. The module with the highest correlation with DMD was MEturquoise in GSE38417 (r=0.97, p=1e−13) and MEturquoise in E-MTAB-2564 (r=0.54, p= 2e−15) as shown in **Figures 5C, D**.

### Common genes and biological functions shared by transcriptome and proteome

**Figure 6:**
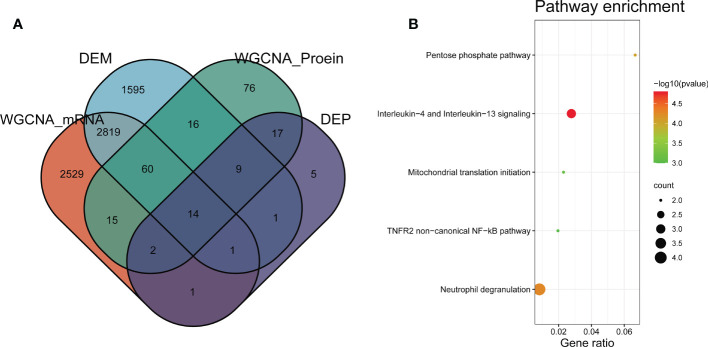
Identification of common genes between transcriptome and proteome in DMD. **(A)** Venn diagram of common genes shared by transcriptome and proteome. **(B)** Pathway enrichment analyses of the overlapping genes.

To discovery functional genes concurrently involved in DMD across transcriptome and proteome, two methods were applied to achieve the key genes. WGCNA was used to identify DMD-related modules. Differential expression analysis was performed to obtain the most dysregulated genes. In total, we identified 14 functional genes as concurrently involved in transcriptome and proteome, including the turquoise module of the GSE38417 (n = 5441), the turquoise module of the E-MTAB-2564 (n = 247), DEMs of GSE38417 (n = 4515), and DEPs of E-MTAB-2564 (n = 55) (**Figure 6A**). Enrichment analysis showed that these genes were mainly enriched in pathways related to Interleukin-4 and Interleukin-13 signaling, neutrophil degranulation, TNFR2 non-canonical NF-κB pathway and pentose phosphate pathway (**Figure 6B**).

### Altered of immune and stromal cell infiltration in DMD skeletal tissue

**Figure 7:**
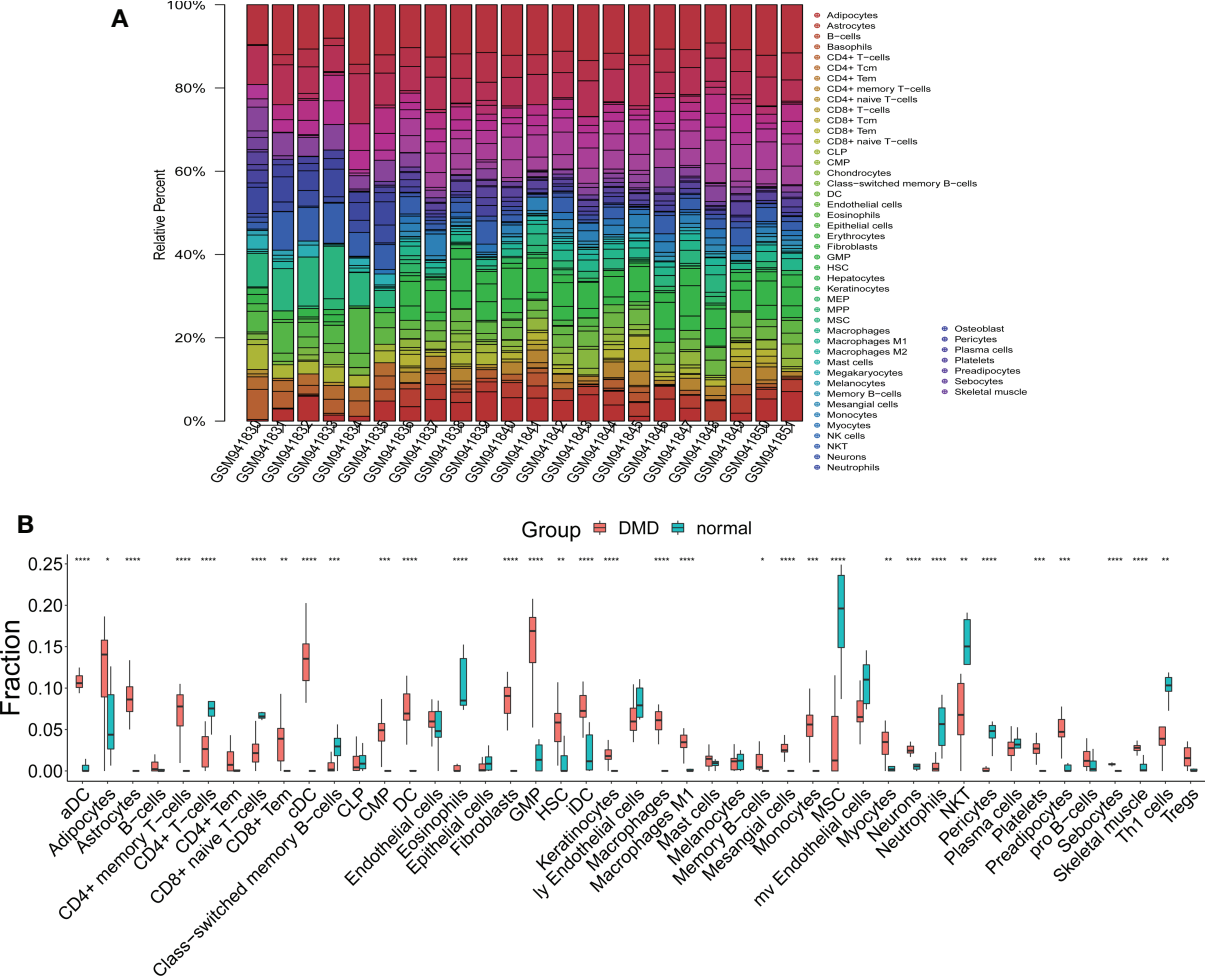
The results of immune and stromal cell infiltration analysis. **(A)** The distribution of infiltrated immune and stromal cells in DMD and normal samples. **(B)** A box plot of infiltrated immune and stromal cells in the DMD and normal groups. *p < 0.05, **p < 0.01, ***p < 0.001, ****p < 0.0001.

We observed 33 significant differences in the infiltration of cells between DMD and control tissue (**Figure 7A**). In DMD, 24 cell types were up-regulated, including aDC, Adipocytes, Astrocytes, CD4+ memory T−cells, CD8+ Tem, cDC, CMP, DC, Fibroblasts, GMP, HSC, iDC, Keratinocytes, Macrophages, Macrophages M1, Memory B−cells, Mesangial cells, Monocytes, Myocytes, Neurons, Platelets, Preadipocytes, Sebocytes, and Skeletal muscle cell (**Figure 7B**). The nine down-regulated cell types were: CD4+ T−cells, CD8+ naive T−cells, Class−switched memory B−cells, Eosinophils, MSC, Neutrophils, NKT, Pericytes, and Th1 cells (**Figure 7B**).

### Correlation analysis between crosstalk markers and infiltrating cells

|r| > 0.7 was set as a strong correlation threshold for further analysis. CTSS was positively correlated with macrophage, DC, macrophage M1 and aDC. VIM was negatively correlated with plasma cells. CD14 was positively correlated with monocytes, DC, and macrophage M1; negatively correlated with D8+ naive T−cells. IFI30 was positively correlated with macrophages. TNFRSF1B was positively correlated with aDC (**Figure 8**).

**Figure 8:**
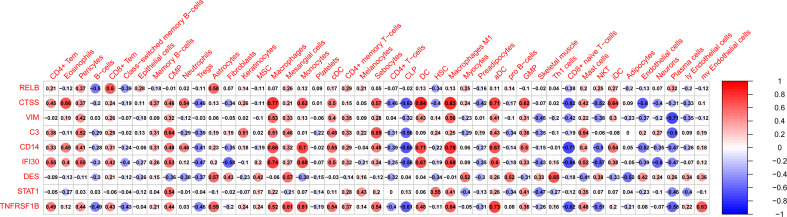
The correlation between hub genes and infiltrated immune and stromal cells. Size of the nodes represents the strength of the correlation between hub genes and infiltrated immune and stromal cells. The larger (or smaller) the nodes, the stronger (or weaker) the correlation. The redder the nodes, the stronger the positive correlation, the bluer the nodes, the stronger the negative correlation.

### TF–miRNA coregulatory network

**Figure 9:**
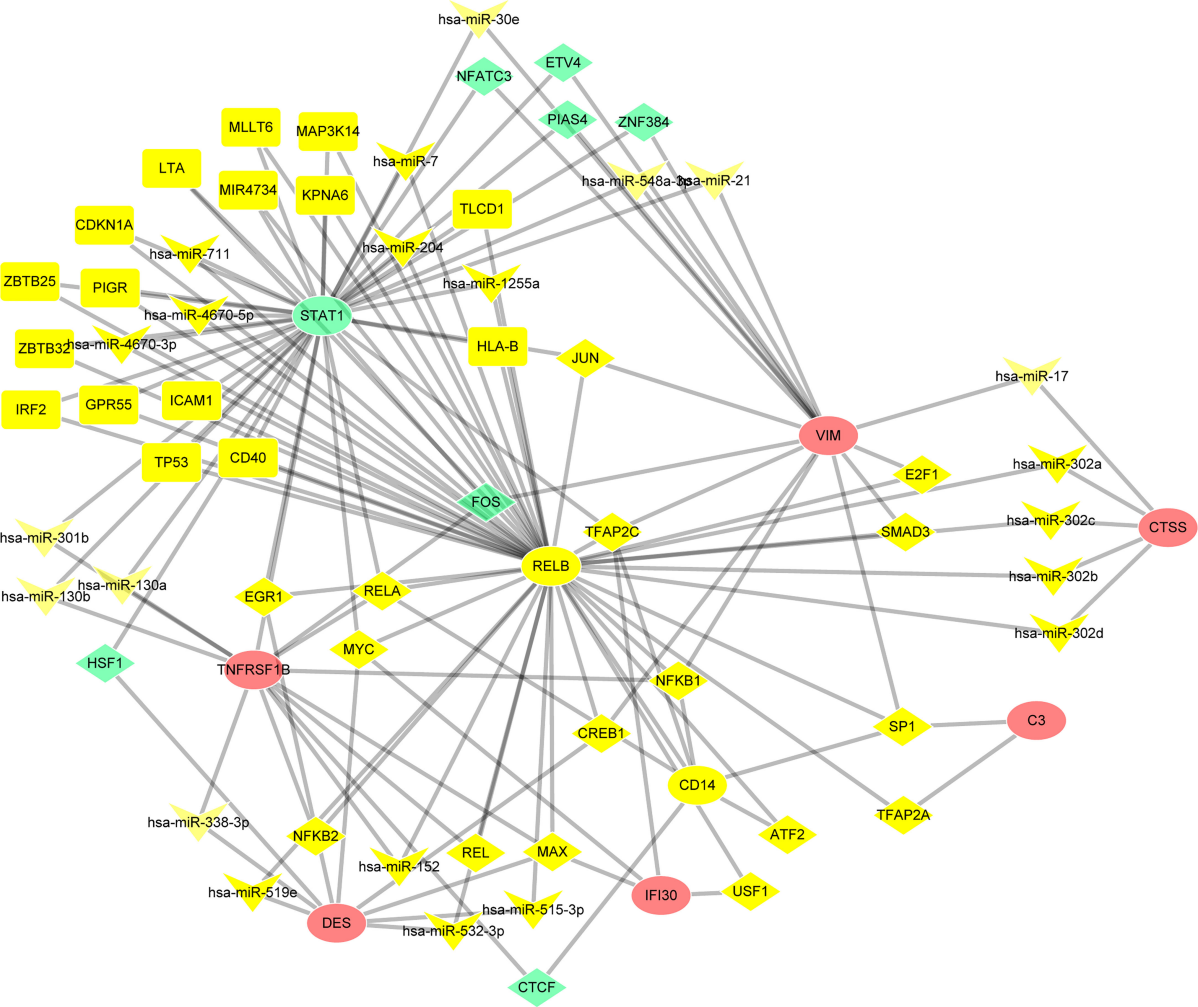
TF–miRNA coregulatory network. The round nodes are validated hub genes, the diamond nodes are TF genes, and the triangle nodes are miRNA genes.

NetworkAnalyst online platform is used to generate TF–miRNA co-regulatory network. The interaction between TFs and miRNAs and the selected hub genes was delivered *via* the TF–miRNA co-regulatory network analysis. This interaction may be the underlying mechanism for the regulation of hub genes expression. The TF–miRNA co-regulatory network is consisted of 70 nodes and 141 edges. A total of 22 miRNAs and 23 TF genes interacted with the validated hub genes, including TFAP2C, MAX, MYC, NFKB1, RELA, hsa-miR-1255a, hsa-miR-130a, hsa-miR-130b, hsa-miR-152, and hsa-miR-17. **Figure 9** shows the TF–miRNA co-regulatory network. RELB owned the largest number of neighbors, followed by STAT1, VIM, TNFRSF1B, DES, CD14, CTSS, IFI30, and C3.

### Construct the signaling information network based on SIGNOR

**Figure 10:**
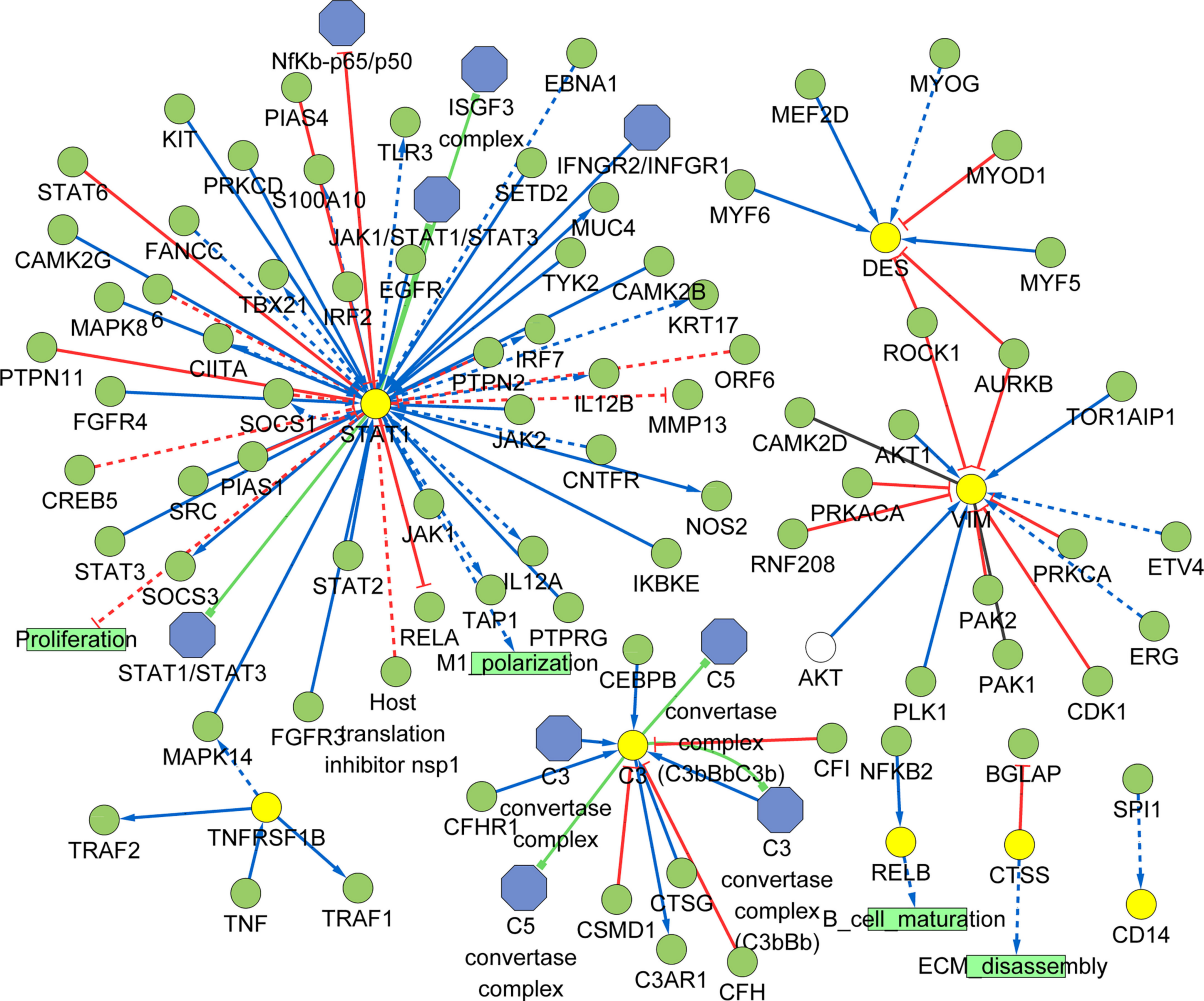
Signaling information network of the hub genes based on SIGNOR.

As shown in the **Figure 10**, we constructed the signaling information network of DEGs based on SIGNOR2.0. STAT1 showed the highest number of interaction, followed by VIM, C3, DES, TNFRSF1B, RELB, CTSS and CD14. Moreover, STAT1 interacted with more complex than other hub genes, including JAK1/STAT1/STAT3, IFNGR2/INFGR1, NfKb-p65/p50, ISGF3 complex. In addition, the analysis also identified the effects of the proliferation, M1 polarization, B cell maturation, and ECM disassembly as potential phenotype, which caused by the alterations in the shortlisted genes. Interestingly, four hub genes (VIM, DES, TNFRSF1B and STAT1) show the interactions through other three genes: AURKB and ROCK1 can directly inhibit VIM and DES; TNFRSF1B can activate STAT1 through increased MAPK14.

### Screening potential diagnostic biomarkers for DMD

**Figure 11:**
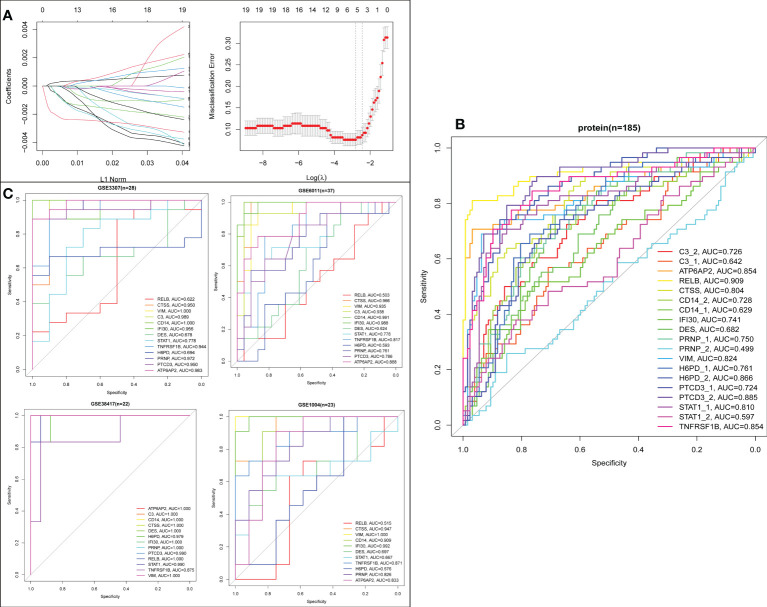
Screening process of the biomarkers for DMD. **(A)** LASSO coefficient profiles of the 19 proteins. **(B)** ROC curve evaluation of the diagnostic effectiveness of the 19 potential biomarkers in protein dataset (n=185). **(C)** ROC curve evaluation of the diagnostic effectiveness of the 19 potential biomarkers in four mRNA datasets.

To establish a LASSO protein signature for diagnosing DMD, 19 intersecting proteins in DEPs and turquoise module from WGCNA were included for LASSO regression analysis in 127 DMD and 58 normal samples. After 10-fold cross-validation, a LASSO model was established, containing ATP6AP2, CTSS, H6PD, PTCD3, and RELB (**Figure 11A**). To determine which intersecting proteins have the diagnose significance of DMD patients, the ROC analyses were conducted to explore the sensitivity and specificity of the intersecting proteins for DMD diagnosis. The results showed that eight proteins (ATP6AP2, RELB, CTSS, VIM, H6PD, PTCD3, STAT1, and TNFRSF1B) presented the best diagnostic value for differentiating the patients with DMD from healthy controls (AUC > 0.8) (**Figure 11B**). We then validated the intersecting genes in the four validation datasets. And four genes (ATP6AP2, CTSS, VIM, and TNFRSF1B) showed excellent diagnostic performance on discriminating DMD in GSE1004, GSE3307, GSE6011 and GSE38417 datasets (all AUC > 0.8) (**Figure 11C**). This indicated that ATP6AP2, CTSS, VIM, and TNFRSF1B could act as biomarkers to estimate the activity of DMD and verify the effectiveness of the treatment of DMD.

### Validation of DMD-associated proteins in DMD patient

**Figure 12:**
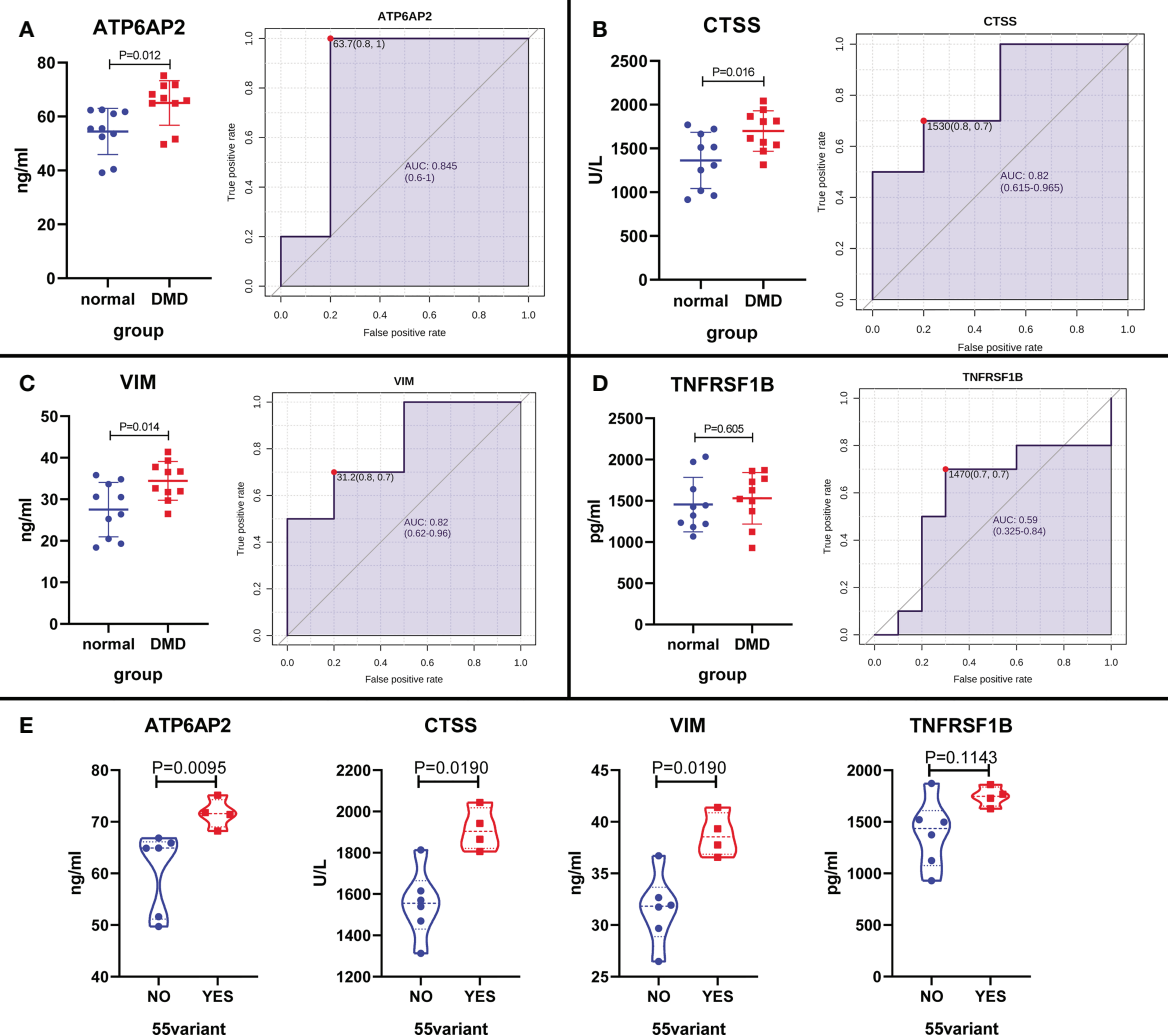
The expression level validation of the four proteins in DMD patients and healthy controls using ELISA. **(A–D)** A boxplot and ROC curve evaluation of the diagnostic effectiveness of the 4 proteins in 10 DMD patients and 10 healthy controls. **(E)** A boxplot evaluation of the diagnostic effectiveness of the 4 proteins in DMD patients with and without exon 55 mutations.

To further increase the reliability of the results, the above proteins (ATP6AP2, CTSS, VIM, and TNFRSF1B) were selected for validation in 10 DMD patients and 10 healthy controls using ELISA. The results showed that ATP6AP2, CTSS, and VIM presented significant higher expression in DMD than healthy controls (P < 0.05) (**Figures 12A–D**). Moreover, the expression of ATP6AP2, CTSS, and VIM in patients with exon 55 mutations was higher than that in patients without exon 55 mutations (P < 0.05) (**Figure 12E**). In addition, LDH was the most related signature between ATP6AP2, CTSS, and VIM expression and characteristics of DMD patients. CRP was the most related signature between TNFRSF1B expression and characteristics of DMD patients (**Supplement Figure 1**). These observations confirmed that the expression levels of ATP6AP2, CTSS, and VIM are highly specific and can be used as sensitive biomarkers for diagnosing patients with DMD, especially for patients with exon 55 mutations.

### Validation of DMD-associated genes in mdx mouse

**Figure 13:**
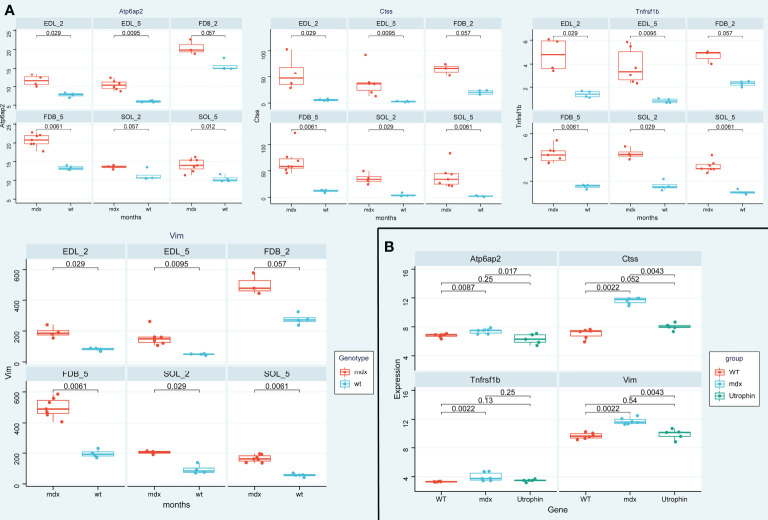
The expression level validation of the four genes in mdx mouse and utrophin groups. **(A)** A boxplot evaluation of the diagnostic effectiveness of the 4 genes in in the three mdx mouse muscles at 2- and 5-month old mice, including the extensor digitorum longus (EDL), the flexor digitorum brevis (FDB), and the soleus (SOL). **(B)** A boxplot evaluation of the diagnostic effectiveness of the 4 genes in utrophin group.

**Figure 14:**
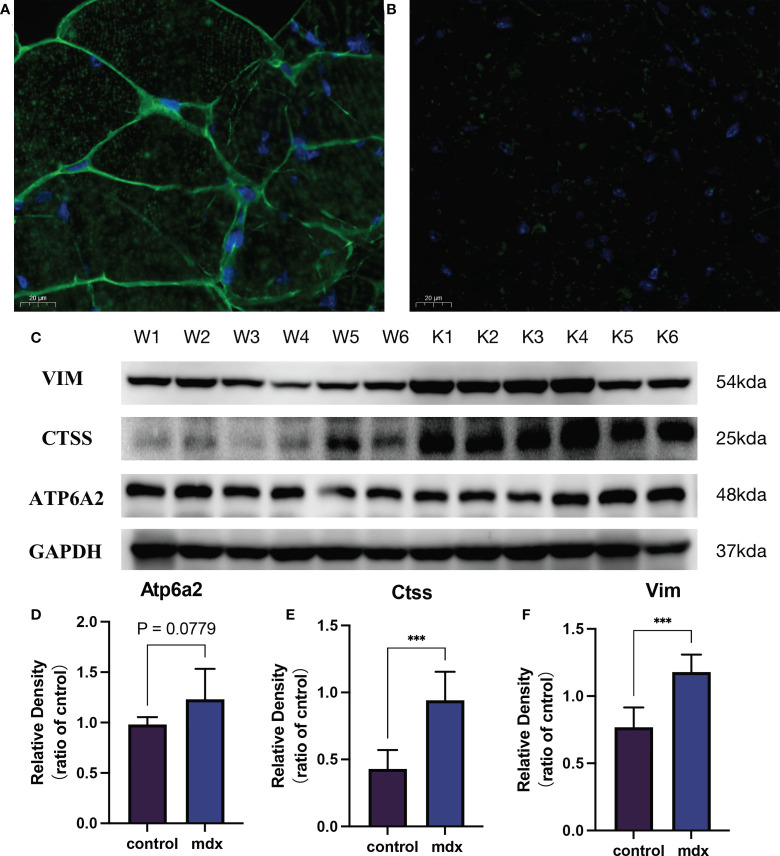
The validation of the biomarkers in the DMD model. Representative images of dystrophin immunofluorescence on wild type **(A)** and mdx mice group **(B)**. **(C)** Representative western blot results of the 3 biomarkers. **(D–F)** Relative protein expression levels of the 3 biomarkers (n = 6). ***P < 0.001, mdx vs. the control group.

To further verify the above results, we first detected the mRNA expression levels of the 4 potential biomarkers in the three mdx mouse muscles, including the extensor digitorum longus (EDL), the flexor digitorum brevis (FDB), and the soleus (SOL). Compared with the sham group, the mRNA expression levels of ATP6AP2, CTSS, VIM, and TNFRSF1B were higher in the three mdx mouse muscles at 2- and 5-month old mice (**Figure 13A**). Since utrophin is highly related to dystrophin and can substitute for dystrophin’s function, we explored the expression levels of these four genes in dystrophin-deficient mice with a transgene expressing high level of full length utrophin. Furthermore, compared with the mdx group, the expression of ATP6AP2, CTSS and VIM recovered in utrophin group (**Figure 13B**). To further verify the results of bioinformatics analysis, we detected the protein expression levels of the 3 potential biomarkers in the muscle tissues. As illustrated in **Figures 14A, B**, the results showed that the DMD model was successfully established. Compared with the control group, the protein expression levels of CTSS and VIM were significant higher in the mdx group (P < 0.001). The changing trend of ATP6AP2 expression was also consistent with the results of bioinformatics analysis (**Figures 14C–F**). Therefore, ATP6AP2, CTSS and VIM may serve as diagnostic and therapeutic biomarkers.

### Establishment and evaluation of the nomogram model

**Figure 15:**
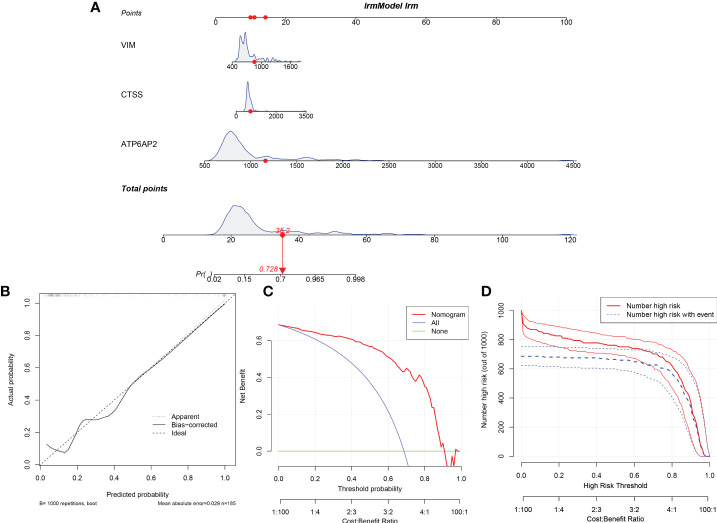
Construction and Evaluation of the nomogram model. **(A)** Construction of the nomogram model based on the 3 potential diagnostic biomarkers. **(B)** Calibration curve. **(C)** Decision curve analysis. **(D)** Clinical impact curve.

Then, the nomogram model was constructed to evaluate the probability of DMD based on the 3 significant diagnostic proteins (ATP6AP2, CTSS and VIM) (**Figure 15A**). The calibration curve suggested brilliant agreement among the apparent curve, bias-corrected curve, and ideal curve (**Figure 15B**). DCA and CIC showed that patients could benefit from the nomogram (**Figure 15C, D**). These results indicated that the prediction efficiency of the model was good.

## Discussion

DMD occurs almost exclusively in males and is inherited in an X-linked recessive pattern. It is a serious degenerative neuromuscular disease, which lead to progressive muscle weakness and weight loss (25). DMD gene, also known as anti-dystrophy protein (dystrophin), is located on the X chromosome. The deletion, repetition or point mutation of the gene makes the muscle cell membrane lose the complete skeleton, resulting in the inability to synthesize normal anti-dystrophy (26, 27). The disease process of DMD is a complex, multi-step process involving multi-factors. It includes destruction of muscle fiber membrane, imbalance of calcium balance, muscle necrosis and exhaustion, accompanied by adipose tissue proliferation and immune cell infiltration (28, 29). Moreover, growing evidence demonstrates the crosstalk between the immune system and the skeletal muscle in inflammatory muscle diseases, including DMD (15, 30, 31). However, these downstream pathways cannot elucidate the pathogenesis of DMD alone. The existing transcriptome and proteome technology make it possible to study many differential expressions of mRNA and proteins at the same time. Therefore, our joint analyses can provide a glimpse of the dynamic regulatory crosstalk between the transcriptome and proteome accompanying DMD, as in immune dysfunction.

In our study, we first profiled mRNA expression in skeletal muscle and protein expression in plasma to identify common differentially expressed mRNAs/proteins. We found that the common dysregulated mRNAs/proteins in DMD were mainly enriched for muscle contraction and inflammation-related pathways. This result is consistent with the fact that DMD is often characterized as a disease of inflammation, due to skeletal tissue activation of the immune response (32, 33). Furthermore, our WGCNA analysis also validated that inflammation pathways were disorder in DMD, such as Interleukin-4 and Interleukin-13 signaling, neutrophil degranulation, TNFR2 non-canonical NF-κB pathway and pentose phosphate pathway. Interleukin-4 can improve the migration of human myogenic precursor cells for the treatment of a damaged myocardium of a DMD patient (34). Additionally, Interleukin-4 and Interleukin-13 triggered the crosstalk between fibro-adipogenic progenitors and anti-inflammatory macrophages, which presented at late stages of regeneration and acted as promoters of myoblast differentiation and fusion (35). Moreover, pentose phosphate pathway dysfunction can contribute to distinct pathological phenotypes in cardiac and skeletal muscle types of DMD (36). Accordingly, the regulatory mechanisms of intersecting genes during DMD were related to inflammation-related biological processes and pathways, which may contribute to a better understanding of the pathologic mechanism of DMD.

Some biomarkers involved in our gene signature have been reported in DMD, but some of them have rarely been investigated in DMD. For example, as a compensatory mechanism for maintaining sarcomere structural integrity, increased vimentin (VIM) is found in dystrophic muscles (37). We demonstrated that VIM exerted increased mRNA and protein expression in DMD, and returned to normal after after utrophin therapy, a substitution of dystrophin. Cathepsin S (CTSS) is a member of cysteine protease family, which degrades damaged and unneeded proteins within the lysosome, and play an key role on immune cells, including in facilitating MHC-II antigen presentation (38). Its induction during muscular dystrophy is a pathologic event that partially underlies disease pathogenesis in mice (39). Consistently, we verified the higher expression in mdx mouse and DMD patients. After utrophin therapy, the expression of CTSS returned to normal level. These results indicated that CTSS may serve as diagnostic and therapeutic biomarkers. ATP6AP2 encodes a protein associated with adenosine triphosphatases (40), which play crucial roles on renin-angiotensin system, energy conservation, and cellular homeostasis (41, 42). However, functions of ATP6AP2 were not explored in DMD. But, previous studies have shown that renin-angiotensin-aldosterone system inhibitors improve membrane stability in dystrophic skeletal muscles (43). Therefore, ATP6AP2 may serve as a new therapeutic strategy through renin-angiotensin-aldosterone system in DMD.

Exonic duplications and deletions within DMD are the main pathogenic variants (44), which has gained special interest as a new therapeutic method for DMD (45). It is reported that DMD exons 45-55 deletion has been postulated as a model that could treat up to 60% of DMD patients (46). However, the associated clinical variability and complications require clarification. In the present study, we proved that the protein expression of ATP6AP2, CTSS, and VIM in DMD patients with exon 55 mutations was higher than that in patients without exon 55 mutations. These results may provide diagnostic biomarkers and crude mechanisms of DMD therapy encompassing exon 55 mutations

## Conclusions

In conclusion, we have innovatively identified ATP6AP2, CTSS and VIM as potential immune-related biomarkers for DMD by combining a bidirectional transcriptome and proteome-driven analysis. Meanwhile, we also constructed a nomogram model based on potential immune-related biomarkers to predict DMD. However, further studies should be employed to prove the above findings. Our work may provide new and valuable insights into the mechanisms and diagnostic value of DMD and its treatments from an immune perspective.

## Data availability statement

The original contributions presented in the study are included in the article/**Supplementary Material**. Further inquiries can be directed to the corresponding authors.

## Ethics statement

The studies involving human participants were reviewed and approved by the Medical Ethics Committee of the first affiliated hospital of Soochow University (No. 2020-145). The patients/participants provided their written informed consent to participate in this study.

## Author contributions

XW and ND designed and conceptualized the report. LY, ML, and JJ collected and analyzed the samples. XW wrote the first draft of the manuscript. QF, HZ, and TT reviewed and revised the manuscript. All authors approved the final manuscript as submitted and agreed to be accountable for all aspects of the work.

## Funding

General Project of National Natural Science Foundation of China (82071300); National Key R&D Program Key Special Project for Intergovernmental Cooperation in International Science and Technology Innovation (2017YFE0103700); Introduction of Clinical Medicine Team Project in Suzhou (SZYJTD201802; Suzhou Gusu Health Talents Program Training Project (GSWS2020002).

## Acknowledgments

We thank Xiaoming Xin for assisting with the experiments.

## Conflict of interest

The authors declare that the research was conducted in the absence of any commercial or financial relationships that could be construed as a potential conflict of interest.

## Publisher’s note

All claims expressed in this article are solely those of the authors and do not necessarily represent those of their affiliated organizations, or those of the publisher, the editors and the reviewers. Any product that may be evaluated in this article, or claim that may be made by its manufacturer, is not guaranteed or endorsed by the publisher.

## Supplementary material

Supplementary Figure 1 Pearson's correlation analysis between the four proteins and the clinical characteristics of DMD.

## Abbreviations

DMD, Duchenne muscular dystrophy; WGCNA, weighted gene coexpression network analysis; GEO, Gene Expression Omnibus; DEMs, differentially expressed mRNAs; DEPs, differentially expressed proteins; ROC, Receiver Operator Characteristic; EDL, extensor digitorum longus; FDB, flexor digitorum brevis; SOL, soleus; DCA, decision curve analysis; CIC, clinical impact curve; VIM, Vimentin; CTSS, Cathepsin S.
