# Supplementary figures and images for: Identification of immune-related features involved in Duchenne muscular dystrophy: A bidirectional transcriptome and proteome-driven analysis
Source: Front Immunol. 2022 Nov 22;13:1017423. doi: 10.3389/fimmu.2022.1017423 (PMC9724784; doi:10.3389/fimmu.2022.1017423)

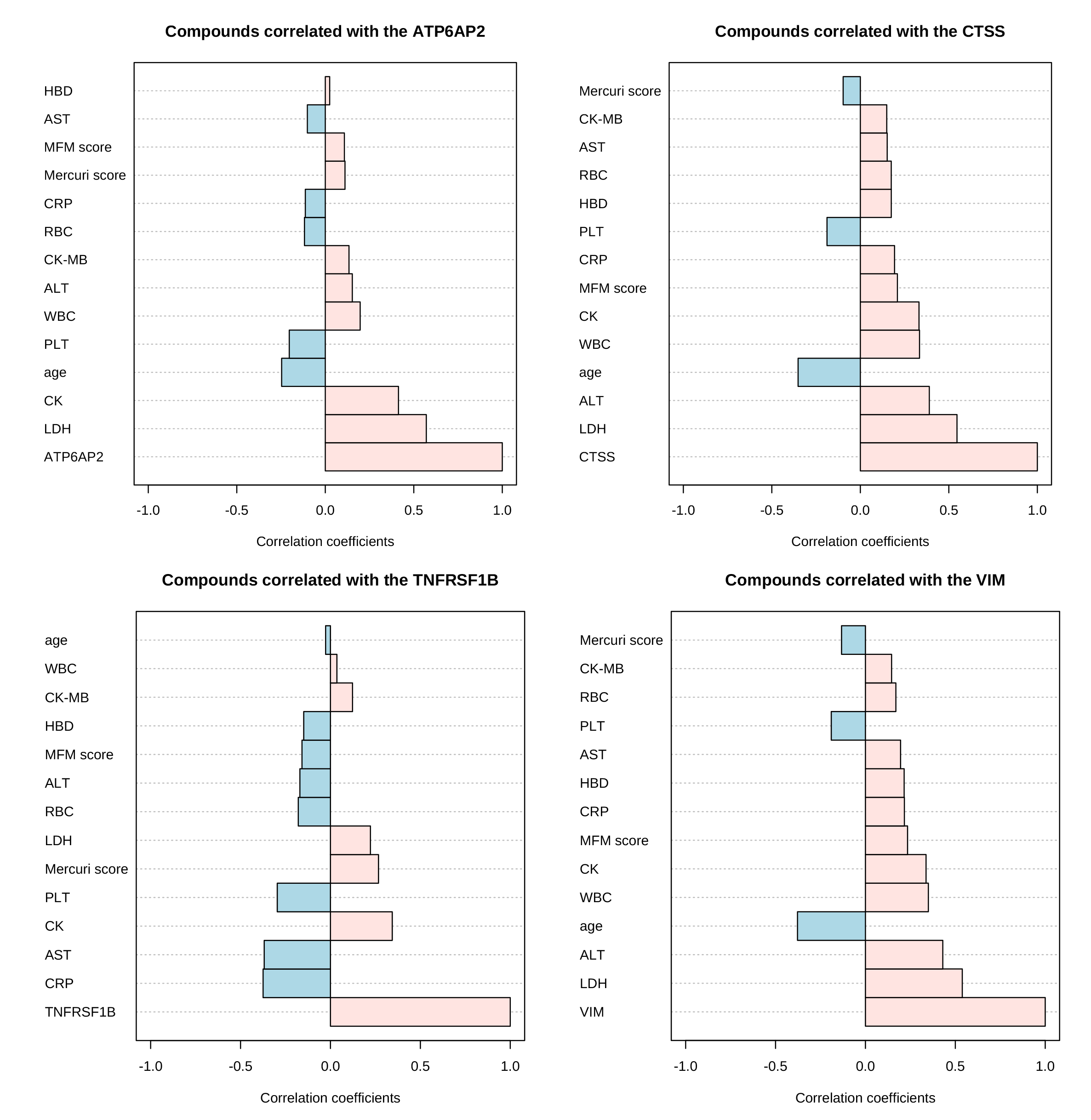

Supplement: Supplementary Figure 1 — Pearson's correlation analysis between the four proteins and the clinical characteristics of DMD. [file Image_1.tif]
