# Supplementary material for: Identification of immune-related features involved in Duchenne muscular dystrophy: A bidirectional transcriptome and proteome-driven analysis
Source: Front Immunol. 2022 Nov 22;13:1017423. doi: 10.3389/fimmu.2022.1017423 (PMC9724784; doi:10.3389/fimmu.2022.1017423)
Supplement: Supplementary file 2 [file Table_1.docx]

Supplement table 1. Characteristics of the DMD patients

| ID | exon 55 mutations | age (year) | WBC (10^9/L) | RBC (10^12/L) | PLT (10^9/L) | CRP (mg/L) | ALT (U/L) | AST  (U/L) | HBD  (g/L) | CK (U/L) | CK-MB (U/L) | LDH (U/L) | MFM score | Mercuri score |
| --- | --- | --- | --- | --- | --- | --- | --- | --- | --- | --- | --- | --- | --- | --- |
| 1 | no | 14 | 8.43 | 5.45 | 275 | 1.67 | 119 | 69 | 511 | 5232 | 88.8 | 635 | 51 | 23 |
| 2 | no | 6 | 11.98 | 4.84 | 284 | 2.45 | 745 | 767 | 1264 | 17802 | 441 | 672 | 92 | 4 |
| 3 | no | 8 | 12.16 | 4.72 | 341 | 2.11 | 367 | 230 | 921 | 9571 | 631 | 1093 | 86 | 17 |
| 4 | no | 14 | 8.25 | 4.92 | 365 | 2.11 | 384 | 418 | 792 | 19828 | 649 | 821 | 42 | 27 |
| 5 | no | 13 | 9.64 | 4.4 | 298 | 1.06 | 245 | 225 | 898 | 27311 | 850 | 178 | 36 | 28 |
| 6 | no | 10 | 8.2 | 5.02 | 259 | 1.18 | 537 | 307 | 843 | 14506 | 240 | 1721 | 68 | 21 |
| 7 | yes | 8 | 6.79 | 4.80 | 285 | 1.03 | 387 | 295 | 628 | 13997 | 7300 | 1461 | 52 | 23 |
| 8 | yes | 7 | 15.87 | 5.41 | 290 | 2.56 | 502 | 199 | 990 | 8986 | 368 | 1590 | 87 | 16 |
| 9 | yes | 6 | 8.72 | 5.01 | 153 | 1.06 | 433 | 209 | 836 | 12606 | 233 | 752 | 90 | 6 |
| 10 | yes | 10 | 12.88 | 5.04 | 275 | 2.38 | 531 | 550 | 912 | 39015 | 104.1 | 2150 | 72 | 20 |

white blood cell count (WBC), red blood cell (RBC), platelet count (PLT), C-reactive protein (CRP), alanine amiotransferase (ALT), aspartate aminotransferase (AST), hydroxybutyric dehydrogenase (HBD), creatine kinase (CK), creatine kinase-MB (CK-MB), lactate dehydrogenase (LDH), Motor Function Measure Scale (MFM) score
